# Identification of core T cell network based on immunome interactome

**DOI:** 10.1186/1752-0509-8-17

**Published:** 2014-02-15

**Authors:** Gabriel N Teku, Csaba Ortutay, Mauno Vihinen

**Affiliations:** 1 Department of Experimental Medical Science, Lund University, Lund, Sweden; 2 Institute of Biomedical Technology, University of Tampere, Tampere, Finland; 3 BioMediTech, University of Tampere, Tampere, Finland

**Keywords:** Protein-protein interaction, Network, Filtering, T cell, TPPIN, Signaling, PPI

## Abstract

**Background:**

Data-driven studies on the dynamics of reconstructed protein-protein interaction (PPI) networks facilitate investigation and identification of proteins important for particular processes or diseases and reduces time and costs of experimental verification. Modeling the dynamics of very large PPI networks is computationally costly.

**Results:**

To circumvent this problem, we created a link-weighted human immunome interactome and performed filtering. We reconstructed the immunome interactome and weighed the links using jackknife gene expression correlation of integrated, time course gene expression data. Statistical significance of the links was computed using the Global Statistical Significance (GloSS) filtering algorithm. P-values from GloSS were computed for the integrated, time course gene expression data. We filtered the immunome interactome to identify core components of the T cell PPI network (TPPIN). The interconnectedness of the major pathways for T cell survival and response, including the T cell receptor, MAPK and JAK-STAT pathways, are maintained in the TPPIN network. The obtained TPPIN network is supported both by Gene Ontology term enrichment analysis along with study of essential genes enrichment.

**Conclusions:**

By integrating gene expression data to the immunome interactome and using a weighted network filtering method, we identified the T cell PPI immune response network. This network reveals the most central and crucial network in T cells. The approach is general and applicable to any dataset that contains sufficient information.

## Background

Cellular interactomes often consist of large numbers of proteins with even larger numbers of connections between them. Typically in protein-protein interaction (PPI) network nodes represent proteins and the links represent relationships between them. This network representation enables the study and visualization of the reconstructed cellular systems.

Data-driven studies on the dynamics of reconstructed PPI networks facilitate investigation and identification of proteins important for a particular process and reduces time and costs of experimental verification [1,2]. Modeling the dynamics of very large PPI networks is computationally very costly. To circumvent this problem, one needs to identify relevant core components of networks without losing vital information. A PPI network constituting most of the relevant core of a cellular system is sufficient to study its dynamic properties [3].

Many methods have been developed to reduce complex directed and undirected networks to their core components. Some of the methods include topological centrality techniques [4], synthetic biology approaches of the minimal gene set of a cell [5,6], complex systems coarse-graining [7,8], and filtering approaches [9-11]. In the centrality methods, topological centrality of nodes is used to identify the non-redundant links and to delete the redundant ones [11]. Minimal gene set approaches aim to identify genes that are crucial for life sustenance and cannot be inactivated under specific optimal growth conditions. These approaches do not take into account interactions between essential gene products [5]. The coarse-graining approaches identify specific motifs in a network, and collapse and replace them by a single node [8]. This process is repeated until there are no more motifs. The final network is less complex but does not consider the structural heterogeneity and broad weight distribution, i.e. the multi-scale nature, of cellular networks.

Network filtering approaches have also been used to reduce network complexity [10-13]. Those that preserve the inherent multiscale structure of natural complex networks have been shown to be better in revealing most of the important components of networks [11,13]. These approaches score the nodes or links, and enable the deletion of those that do not deviate significantly from a null model.

In this study, we identified the network of proteins relevant in T cells by filtering the immunome interactome using the result from Global Statistical Significance (GloSS) [13] algorithm and a constraint of connectivity of the T cell receptor (TCR) signaling pathway. We compiled genes for the major immune processes and used them to reconstruct the immunome interactome, i.e., all the PPIs of the immunome. We then integrated gene expression profiles for the corresponding genes across several experiments. Jackknife correlation for gene expression was then used to weigh links between the proteins encoded by the genes. To maintain the multiscale structure of the network during filtering, we used the GloSS algorithm. This algorithm utilizes a global null model of the link weight and the degree distribution of the network. It computes the statistical significance for each link. For the null model, GloSS assigns weights from the weight distribution of the network, independently and randomly, without changing its topology. We filtered the network by deleting links based on their p-values (computed by GloSS) in descending order. To determine the endpoint of the filtering, we imposed as a constraint, the existence of a single path between the components of the NF-κB and TCR complexes.

Because we investigated the global and aggregate characteristics of the system and integrated T cell gene expressions, we can assume that the filtered network contains most of the components central for T cell signaling [14]. This was supported by Gene Ontology (GO) and essential genes enrichment analysis.

## Results

### Protein-protein interaction network

We used altogether 1579 proteins for the network filtering (Additional file 1). Eight hundred and eighty five human immunome genes were obtained from the Immunome Knowledge Base (IKB) [15]. As IKB contains only the most essential immunome genes and does not necessarily contain full pathways, it was supplemented with proteins for key immune system pathways derived from the KEGG Pathway database [16] (Table 1).

**Table 1:** KEGG pathways used to supplement IKB dataset

**KEGG identifier**	**Name of KEGG pathway**
path:hsa04010	MAPK signaling pathway
path:hsa04062	Chemokine signaling pathway
path:hsa04514	Cell adhesion molecules
path:hsa04612	Antigen processing and presentation
path:hsa04620	Toll-like receptor signaling pathway
path:hsa04621	NOD-like receptor signaling pathway
path:hsa04622	RIG-1-like receptor signaling pathway
path:hsa04630	Jak-STAT signaling pathway
path:hsa04640	Hematopoietic cell lineage
path:hsa04650	Natural killer cell mediated cytotoxicity
path:hsa04660	T cell receptor signaling pathway
path:hsa04662	B cell receptor signaling pathway
path:hsa04664	FcϵRI signaling pathway
path:hsa04666	FcγR-mediated phagocytosis
path:hsa04670	Leukocyte trans-endothelial migration
path:hsa04672	Intestinal immune network for IgA production
path:hsa04610	Complement and coagulation cascades
path:hsa04623	Cytosolic DNA-sensing pathway

The protein products of the genes that take part in these pathways were used to supplement the protein data from the IKB database. The combined protein data represent the immune response protein dataset.

**Figure 1:**
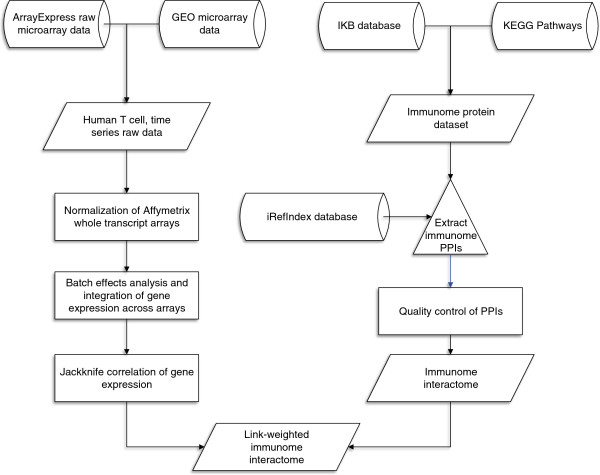
**Workflow for the reconstruction of the immunome interactome.** The general steps taken to reconstruct the immunome interactome are shown. The cylinders represent data repositories from which data was retrieved. Parallelograms represent data, either retrieved from databases or obtained by analyses. Rectangles represent performed analyses. T cell microarray experiments available in ArrayExpress and the GEO databases were retrieved. These experiments included at least 3 samples. The selected experiments were normalized using the R/Bioconductor libraries. Batch effect analysis was done and all experiments were merged or integrated. Jackknife Pearson correlation coefficient was calculated for the integrated dataset. Immunome proteins were retrieved from the Immunome knowledge Base (IKB) and the KEGG pathways databases. Major immune response pathways from the KEGG were used to supplement the IKB immunome proteins. Immunome interactome was obtained by retrieving PPIs for the immunome protein dataset from the iRefIndex database. To reduce noisy PPIs we used the ppiTrim method and further filtered its output of redundant and non-immune response PPIs. The Jackknife correlation coefficients were used as link weights.

The PPI network was reconstructed for the immunome proteins (see workflow in Figure 1). PPI data were retrieved from iRefIndex database (version 9.0) which compiles PPIs from the major repositories [17]. ppiTrim (version 1.2.1) was used for general filtering according to Stojmirovic et al. [18]. Only experimentally verified and binary PPIs were retained. Moreover, multiple binary PPIs encoded by the same gene pair were collapsed into a single PPI. Finally, binary interactions to proteins outside the immunome were eliminated. A total of 5603 PPIs between 1259 immunome proteins were available after these pre-processing steps (Additional files 2 and 3).

### Gene expression correlation

**Figure 2:**
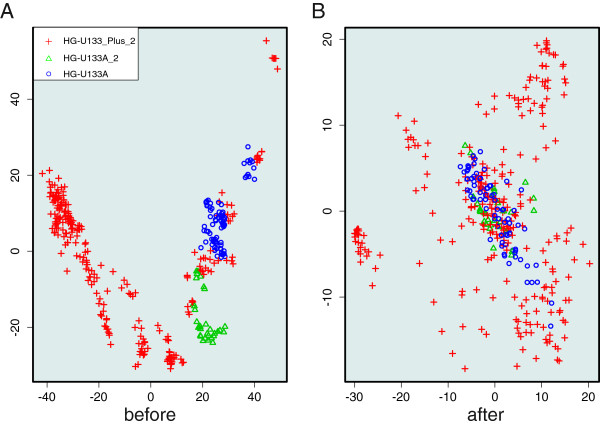
**PCA analysis of normalized gene expression data before and after batch effect analysis. A**. PCA before batch effect analysis. Experiments from the different platforms cluster together. **B**. PCA after batch effect analysis. Results for experiments from different platforms overlap. The platforms are indicated by symbols.

T cell gene expression datasets were obtained from NCBI GEO [19] and EBI ArrayExpress [20] databases. Altogether 16 time series datasets (Additional file 4) containing 384 samples derived from 5 platforms fulfilled the set criteria. After pre-processing, batch effect analysis was performed. Further, exploratory Principal Component Analysis (PCA) was done to examine the effect and performance of the batch effect analysis (Figure 2). The samples cluster according to experiment and platform before batch effect analysis. However, after batch effect correction, samples performed on all three platforms overlap with each other. The batch effect-corrected expression data were integrated or merged together. Of the genes encoding the 1259 immunome proteins, 1149 were expressed in at least 80% of the samples in the merged dataset and were thus included in the analysis.

Next, the mean of the jackknife Pearson product-moment correlation coefficient was calculated for the pre-processed and merged expression values for all gene pair combinations. In total, 1140 genes representing 5164 gene pairs encoding interacting proteins in the immunome interactome were used for further analysis.

**Figure 3:**
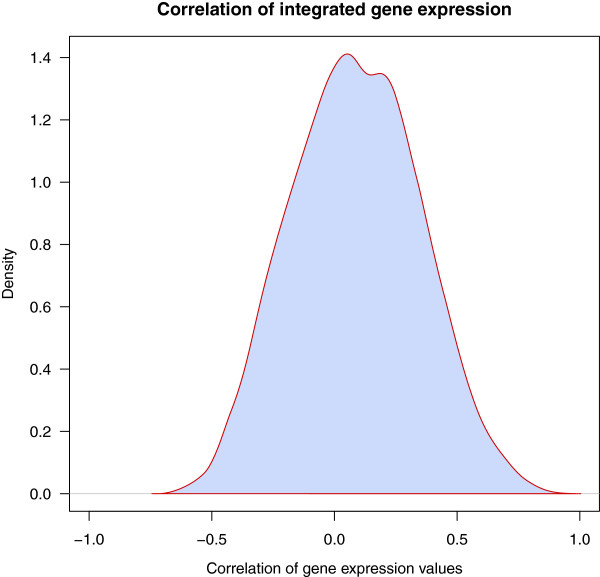
**The distribution of the jackknife correlation of gene expression among the immunome proteins.** Density plot for the distribution of gene expression correlation of 1140 immune response-related genes used in this study. Gene expression values were normalized, and integrated across experiments after batch effect analysis. The correlation coefficient for each gene pair was derived by the jackknife Pearson correlation coefficient across all the integrated microarray samples. A major part of the gene expression correlation values is between -0.5 and 0.5.

The distribution of the integrated jackknife correlation values is shown in Figure 3. The maximum gene expression correlation is 0.88, between *ITGA2B* (integrin α-IIb or *CD41*) and *ITGB3* (integrin β-3 or *CD61*). The encoded proteins form an integrin receptor complex [21] and are thus co-expressed. Their functions include cell adhesion, cell-cell interaction, receptor for several molecules and platelet activation [21]. The minimum correlation of -0.62 was observed between *LCK*, coding for lymphocyte-specific protein tyrosine kinase, and *PAK2*, p21 protein (Cdc42/Rac)-activated kinase 2. LCK is an important signaling protein in many cellular processes, especially in T cell receptor (TCR) activation and T cell development [22]. PAK2 is a member of the PAK proteins (a family of serine/threonine kinases) targeted by small GTP proteins, CDC42 and RAC1 [23,24]. They take part in several signaling pathways, including the TCR signaling network. Albeit association of increased PAK2 activity in cells that overexpress Src kinases, PAK2 and LCK have not been shown to directly interact with each other [25]. The mean of the correlation values for all gene pairs is 0.09 and most of the correlation coefficients lie between -0.5 and 0.5.

### T cell-specific PPI network

We reconstructed the immunome PPI network as a weighted and undirected graph. The nodes, links, and link weights of the graph represent, respectively, the immunome protein coding genes, the PPIs and the absolute value of the mean jackknife expression correlation between the connected immunome protein coding genes.

The topology and weight distribution of naturally occurring complex weighted networks are heterogeneous and tightly connected. This makes the identification of the relevant structure that maintains the multiscale nature of the network nontrivial. Thus, we used the GloSS algorithm [13] to compute a p-value, for each link. GloSS identifies the relevant backbone of a weighted graph while retaining the multiscale coupling of its weight distribution and topological characteristics. It uses a global null model that describes both the structure of the network and its weight distribution. The p-values computed by GloSS were used to filter the network by deleting links based on their p-values, in descending order. We monitored the filtering process to make sure that the central networks between TCR, and NF-κB and NFAT signaling pathways remained intact. These pathways have been shown to be crucial for T cell signaling [26,27] and therefore cannot be disconnected without destroying essential cellular processes.

**Figure 4:**
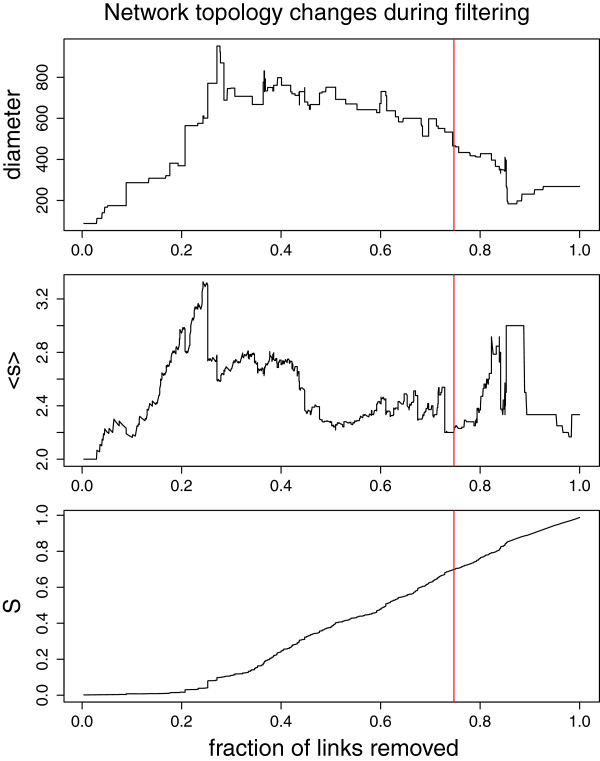
**Network topology changes during GloSS-, NFAT- and NF-κB-assisted filtering.** The immune response PPI network topological changes during the filtering process. Network measures were used to investigate the immunome interactome during filtering. The x-axis in each panel is the fraction of nodes removed during filtering. On the y-axis, the top panel shows changes in the network diameter, the middle panel changes in the average size of the isolated components excluding the largest or giant component (<s>). The bottom panel shows changes in the relative size of the largest or giant component (S). The relative size of the largest component is the number of nodes in the largest component divided by the number of nodes in the whole network. That is, *n*_*rel*_ = *n*/*N*, where *n*_*rel*_ is the relative size of the largest component, *n* is the number of nodes in the largest component and *N* is the number of nodes in the whole network). Each of the network measures were plotted against the fraction of links removed during filtering. The vertical line shows the point at which the paths between the TCR complex and the NF-κB and NFAT downstream components are broken. This also represents the point at which the filtering process stops. This indicates that the connectivity and robustness of the filtered network at this endpoint is maintained. Thus the connectivity and robustness inherent in the immunome interactome is maintained in the TPPIN.

**Figure 5:**
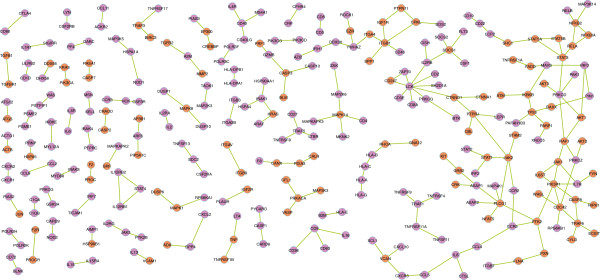
**T cell PPI Network and essential genes enrichment.** After removing non-immunome genes and immunome genes without the lethality annotations, we calculated the hypergeometric distribution and Fisher’s exact test for significance. The figure demonstrates the enrichment of essential genes across almost every component of the TPPIN and for the whole network (p-value = 1.37 × 10^-10^). The node labels are gene identifiers for the genes coding the proteins. The nodes colored red represent essential genes. The essential genes data is based on the human orthologs of the mouse lethality genes from the Mouse Genome Informatics database.

We followed changes of structural and biological features in the PPI network during the filtering process with network parameters. The diameter of the network represents the longest minimum distance between the nodes. We used as measures the changes in diameter, the relative size of the largest connected component and the average size of the isolated components [28]. These network topology scores show how connectivity, integrity and robustness of the network are changed when links are removed during the filtering process (Figure 4). All the panels in Figure 4 indicate that at the cutoff point most of the remaining network’s connectivity and integrity is still maintained. We call the remaining network the T cell PPI Network, TPPIN (Figure 5). TPPIN consists of 288 nodes, 227 links in 73 connected components (Table 2).

**Table 2:** General structure of the T cell PPI network

**Number of nodes in connected component**	**Number of links**	**Number of components in the network**
91	100	1
14	14	1
6	5	2
5	4	3
4	3	5
3	2	13
3	3	1

A component represents a set of nodes that are all connected to each other, either directly or indirectly. Components with two nodes are not included in the table.

### Correlation distribution before and after filtering

**Figure 6:**
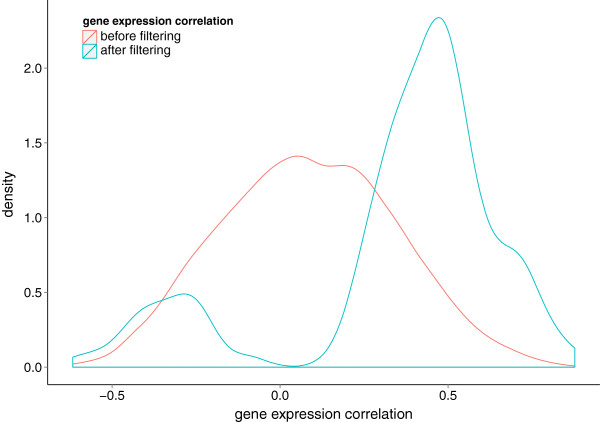
**Distribution of correlation coefficient before and after filtering.** Threshold algorithms filter a network by removing edges whose weights are below an arbitrary cutoff. Such a network would have lost its multiscale structure and thus its core structure. We probe the distribution of the gene expression correlation coefficient to establish whether the multiscale structure of the immunome interactome is retained in the filtered T cell PPI network. The red and blue curves represent, respectively, the distribution of gene expression correlation before and after filtering. The filtering approach preserves a broad distribution of link weights, i.e., most with large weights and some with small weights.

Threshold algorithms filter a network by removing edges whose weights are below an arbitrary cutoff. Such a network loses its multiscale and, thus, its core structure. We probed the distribution of the gene expression correlation coefficient to establish whether the multiscale structure of the immunome interactome is retained in the filtered T cell PPI network (Figure 6). The filtering process succeeds in maintaining not just the links with large weights but also links with lower weights. Thus, the filtering process maintains the multi-scale structure of the network and retains edges that are crucial for the T cell PPI network.

### Effect of noise on the filtering procedure

To test the sensitivity of our filtering procedure to noise we introduced randomness to the immunome interactome, before performing filtering, by randomizing fractions of the link weights while preserving the topology of the network. We refer to these networks as the Link Weight-Randomized Networks (LWRNs). Nine such networks were created based on the fraction of weights randomized. Thirty iterations were conducted for each LWRN. Each iteration consists of choosing randomly a fraction of links, reassigning their weights randomly, conducting the filtering procedure, and calculating network topology statistics. The topology features calculated for each iteration include node degree, average path length, betweenness centrality of both the nodes and the links, clustering coefficient of the network, and the intersection between the TPPIN and the LWRN. These measures indicate the local and global connectivity of a network. We retained the average of the above quantities.

**Figure 7:**
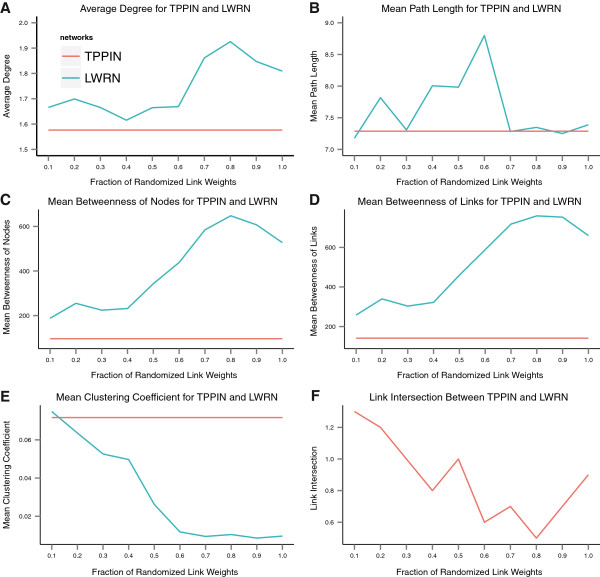
**Comparison of T cell PPI network and link weight-randomized networks.** Robustness analysis on the filtering procedure was performed by randomizing the immunome interactome link weights to introduce noise. Thirty iterations were conducted so that a fraction of links are selected randomly and their weights randomly reassigned for each iteration. The Link Weight-Randomized Network (LWRN) was filtered and node degree, average path length, betweenness centrality of both the nodes and the links, clustering coefficient of the network, and the intersection between TPPIN and the LWRNs, were calculated. The blue line curves are for the LWRNs and red lines for the TPPIN. **A**. Change in average degree, **B**. change in mean path length, **C**. change in mean betweenness of nodes, **D**. change in mean betweenness of links, **E**. change in mean clustering coefficient, **F**. Link intersection between TPPIN and LWRNs.

Figure 7 shows the similarity or dissimilarity between TPPIN and LWRNs. Figure 7 A-E, shows that as more of the link weights are randomized, the topology of the LWRNs diverges significantly from TPPIN. Moreover, as Figure 7 F shows, there is very little overlap of links between the LWRNs and TPPIN.

### Gene Ontology over-representation and semantic similarity analysis

GO term over-representation analysis was performed for the TPPIN proteins and shows that, at level two details, most of the biological process terms are relevant for T cell function (Table 3 and Additional file 5). For example, the term *positive regulation of lymphocyte activation pathway* (GO:0051251, p-value = 9.74 × 10^-7^), *regulation of immune response* (GO:0050776, p-value = 1.11 × 10^-6^), and *intracellular protein kinase cascade* (GO:0007243, p-value = 3.40 × 10^-6^) terms are among the most significantly enriched after adjusting for multiple comparisons. In addition to significant immune response-related terms, there are also those for general cellular processes.

**Table 3:** **GO *biological process *** **term enrichment for TPPIN**

**GO ID**	**Term**	**Number of significant vs. annotated genes**	**Expected number of genes**	**Raw vs. adjusted P value**
GO:0051251	positive regulation of lymphocyte activation	128/61	32.51	5.40 × 10^-09^/9.74 × 10^-07^
GO:0043067	regulation of programmed cell death	289/114	73.41	4.77 × 10^-10^/4.60 × 10^-07^
GO:0050776	regulation of immune response	313/118	79.51	6.79 × 10^-09^/1.11 × 10^-06^
GO:0048523	negative regulation of cellular process	401/142	101.86	1.05 × 10^-08^/1.62 × 10^-06^
GO:0050867	positive regulation of cell activation	144/64	36.58	7.04 × 10^-08^/5.59 × 10^-06^
GO:0048584	positive regulation of response to stimulus	401/140	101.86	5.10 × 10^-08^/4.40 × 10^-06^
GO:0042981	regulation of apoptotic process	285/113	72.39	4.04 × 10^-10^/4.60 × 10^-07^
GO:0007243	intracellular protein kinase cascade	330/121	83.82	3.13 × 10^-08^/3.40 × 10^-06^
GO:0019221	Cytokine mediated signaling pathway	163/73	41.40	3.85 × 10^-09^/8.07 × 10^-07^
GO:0006468	protein phosphorylation	313/116	79.51	3.63 × 10^-08^/3.51 × 10^-06^

The “universe” is the immunome protein data and the enrichment is for the filtered immunome interactome, the T cell PPI network (TPPIN).

To better investigate the similarity or difference between the immunome interactome and the TPPIN network, we explored semantic similarity of the networks using the GOSemSim package available from R/Bioconductor. The semantic similarity ranges between 0 and 1. The similarity between the immunome interactome and TPPIN proteins in the biological process and molecular function terms were very high, i.e., 0.91 and 0.92, respectively, indicating that the TPPIN is very representative of the immunome interactome.

### Essential genes over-representation analysis

Essential genes are indispensable to the survival of a cell or organism. To account for how essential the genes are, we performed an over-representation analysis to identify the proportion of the essential TPPIN genes. We conducted a hypergeometric test on the human orthologs of the mouse lethality genes from the Mouse Genome Informatics resource [29]. The results show a highly significant enrichment of essential genes in the TPPIN (p-value = 1.37 × 10^-10^, Table 4 and Figure 5).

**Table 4:** Essential genes overrepresentation

	**Number of genes**	**Number of genes annotated in MGI**	**Number of lethality genes annotated in MGI**^**a**^	**Expected number of lethality genes in MGI**	**P-value for hypergeometric test**
Immunome interactome	1140	949	312		
TPPIN	288	256	105	59	1.37 × 10^-10^

The T cell PPI network is the resulting network after filtering the immunome interactome. MGI^a^ is the Mouse Genome Informatics database.

### Interconnection of T cell-specific pathways

**Figure 8:**
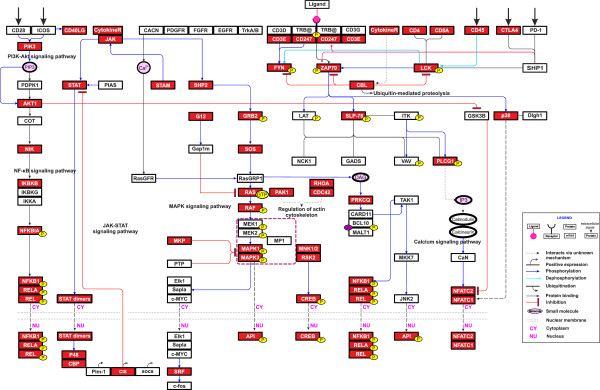
**T cell PPI Network mapped to TCR, with main JAK-STAT and MAPK signaling pathways.** The figure shows the TCR and the main JAK-STAT and MAPK signaling networks that are connected to the TCR-dependent T cell response. The pathway information is adapted from the KEGG Pathways resource. The TPPIN proteins are represented by red-colored boxes with protein names in white text. The signaling network demonstrates the effectiveness of the filtering approach as many of the central proteins in T cell response are left intact after filtering.

The TPPIN proteins were mapped onto the TCR, JAK-STAT and MAPK signaling pathways that are central for T cell functions [30] (Figure 8). Albeit containing just a third of the proteins in the initial network, the TPPIN includes almost all the main components for the remaining pathways. Except for CD3γ and CD3δ, all the CD3 proteins of the TCR complex are present in the TPPIN. Further, most proteins important for early T cell activation, NFAT, AP1, NF-κB, T cell co-inhibitory and co-stimulatory signal transduction are present. Overall, most of the proteins in the important pathways for T cell signaling are present in the TPPIN. This indicates that the filtering procedure was able to, first of all, identify central pathways and, secondly, to keep their connectivity. As a novel feature the TPPIN indicates the interconnection of the central pathways.

## Discussion and conclusions

In this study, we identified the network of proteins relevant for T cells by filtering the multiscale immunome interactome using the GloSS filtering algorithm [13]. We compiled the genes for the major immune processes and reconstructed the immunome interactome. Then we integrated gene expression profiles across several gene expression experiments. The jackknife correlation for gene expression was used to weigh links between the proteins encoded by the genes. Next, we used the output from GloSS to filter the network. The filtered network contains most of the relevant T cell functional components and was designated TPPIN. This was confirmed by the overrepresentation analysis conducted with GO terms and essential genes.

Many important components of the TCR-dependent signaling pathways are present in the TPPIN. Except for CD3γ and CD3δ, other components of the TCR complex which are included in the microarrays used in this study, are present (TCR-α and -β are not present in the microarrays). The co-receptors CD4 and CD8 are both present, as well as, all the proteins that make up the immunological synapse. With the exception of LAT, GADS and ITK, most proteins that are central in the immediate TCR receptor-associated intracellular signaling after the formation of the immunological synapse and TCR activation are present in the TPPIN, including LCK, FYN, CD45, ZAP70, SLP-76 and PLC-γ.

After its activation, PLC-γ cleaves PIP2 into the second messenger IP3 and DAG [31,32]. This event sets off the activation of three important signaling pathways in T cells that end up with transcriptional activation of NFAT, NF-κB and AP-1 [30]. DAG activates PKC-θ, which in turn activates NF-κB [33]. IP3 activates CaN through the calcium signaling, and CaN subsequently activates NFAT [34]. DAG activates RasGRP [35,36], which in turn initiates the activation of the MAP kinase cascade [37], culminating in the activation of FOS [38]. Key proteins in the NF-κB pathway including PKC-θ, IKK-β and IκB [39] are present in the TPPIN. With the exception of RasGRP, MEK1/2 and ELK co-complexes, the other vital proteins in the MAP kinase signaling cascade [40] and the JAK-STAT pathway [41] are captured by the TPPIN. These results show how the TPPIN represents relevant T cell-related parts of the immunome interactome.

During the filtering step the central networks connecting the TCR complex to the NF-κB and NFAT signaling pathways were kept intact. Although the NFAT and NF-κB pathways are present in many different cell-types, they are central for T cell survival and functions. The connectivity of these components was used to determine the end point for the filtering process. The filtering was continued until there was a minimum number of links, i.e., one, between the TCR, and NF-κB and NFAT components.

GO term enrichment analysis confirms that several of the TPPIN proteins have important T cell functions. As an example of biological process term enrichment, the *positive regulation of lymphocyte activation pathway* (GO:0051251), *regulation of immune response* (GO:0050776), and *intracellular protein kinase cascade* (GO:0007243) terms are significantly enriched. To further probe the similarity between the immunome interactome and the TPPIN proteins we calculated their semantic similarity with respect to biological process and molecular function GO terms. The networks were semantically very similar in both types of GO terms. Because essential genes are indispensable for the survival of a cell, their enrichment in the cellular network would indicate that the network is crucial to the cell. Thus, we investigated the enrichment of essential genes in the TPPIN. The analysis showed a highly significant enrichment of essential genes in the TPPIN. These independent lines of evidence support the applicability of the network filtering routine.

Due to the scarcity of time course microarray experiments with uniform design, gene expression datasets with different designs were used. Integrated analysis was carried out to identify and exclude biased datasets [42,43]. The normalization and batch effect analysis steps served to considerably minimize the effect of bias for correlation calculation from the experimental studies.

Global and aggregate cellular interactions are more plausible between proteins encoded by co-expressed genes than between gene products whose expression patterns are uncorrelated [14]. Since we investigated the global and aggregated characteristics of the immune response in T cells by integrating gene expression experiments conducted for T cell lines, the correlation coefficients represent the aggregate strength of the T cell-specific relationship between the genes and their interacting protein products [14,44].

To explore the changes in the network during the filtering process we investigated changes in the diameter, relative size of the largest component and the average size of the connected components of the network. These network measures have been shown to indicate the connectivity status of a network and its robustness against link removal or loss [28,45]. The changes in network statistics during the filtering process showed that TPPIN maintains most of the integrity and connectivity of the immunome interactome.

Certain aspects of T cell function have been previously modeled [46-49]. Most of these studies are related to gene regulatory networks and modeling of small signaling networks involving transcription factors and their targets, selected to include genes or proteins well-known in the modeled system. In these studies, the typical number of genes or proteins is in a few tens, whereas we started with the entire immunome interactome of 1149 proteins and 5164 links, and ended up with a core network that contains 288 proteins and 227 links. The number of nodes and links in the TPPIN makes it amenable to tailored cellular systems modeling and experimental studies. Our approach is unsupervised and does not utilize any preconceptions, yet, it reveals the central proteins and their networks.

The filtering process carried out in this study has some potential limitations. It needs several time course expression datasets for the cell-type or tissue of interest and each experiment should consist of at least 3 samples. A set of proteins is needed to track the connectivity of the vital pathways and a stop criterion when key pathways are broken. However, these limitations are not of great practical importance in the present era of high throughput studies.

The reported filtering routine can capture the core cell-type-specific PPI network for any cell-type from time series gene expression datasets, and is not limited to well-known systems. The approach opens ways for modeling protein interaction networks of cellular systems, even when pathways are not previously well characterized.

## Methods

### Protein-protein interaction network reconstruction

Human immunome proteins were obtained from the IKB [15] and supplemented with key immune system pathways from the KEGG pathways database [16].

Experimentally verified and consolidated PPI data for the human immunome proteins was retrieved from the iRefIndex database version 9.0 [17]. First, the ppiTrim version 1.2.1 [18] was used to filter the iRefIndex dataset. This algorithm maps protein interactants to NCBI gene identifiers and removes undesired raw interactions, deflates potentially expanded complexes, and reconciles annotation labels from the different PPI databases. Second, non-experimentally verified, non-human, complex and self-self PPIs were omitted. Third, we collapsed multiple binary PPIs whose interactants are products of the same genes. Finally, we eliminated PPIs for which both interactants were not immunome proteins (Figure 1). The igraph library [50] in the R statistical programming environment [51] was used to reconstruct and analyze the PPI network. Visualizations were done using Cytoscape version 2.8 [52].

### Gene expression data

We retrieved microarray time course datasets for human T cell-lines from GEO [19] and ArrayExpress [20] databases. Each experiment had to contain at least three samples and at least one for time zero for baseline data. GEO datasets that consisted of samples from multiple platforms were split into multiple experiments, so that each experiment consisted of samples for the same microarray platform. To reduce bias during gene expression integration across experiments we included only experiments performed on Affymetrix whole transcript array platform U133A, U133A 2.0, U133B, U133 plus 2.0 and U95A arrays.

### Pre-processing of gene expression data

R and Bioconductor libraries were used for data pre-processing [51,53]. The raw data for each gene expression dataset was retrieved. Pre-processing consisted of quality control using boxplots, arrayPLM and simpleaffy routines. For each experiment, samples were normalized using default parameters of the Robust Multi-Array algorithm [54] implemented in the affy library [55]. To convert probe sets to gene expressions, we used the mean of the probe sets to represent the corresponding gene’s expression using the platform-dependent libraries in the Bioconductor project [56]. Gene expressions for non-protein coding genes in the immunome protein dataset were removed.

The gene expression datasets were merged and batch effects were analyzed. We also performed PCA analysis before and after batch effect analysis to examine its effect and performance on the normalized datasets. The batch effects and PCA analysis were performed using the ComBat [43] and plotMDS algorithms implemented in the inSilicoMerging library [42] in Bioconductor.

### Gene expression correlation

The mean of the jackknife Pearson correlation coefficient of the merged and pre-processed expression values for all gene pair combinations was calculated using the bootstrap library implemented in R. These correlation values were converted to absolute values and used as link weights for the immunome interactome.

### Protein network filtering

We reconstructed the immunome PPI network as a weighted and undirected graph using the igraph package in R. The nodes, links, and link weights of the graph represent, respectively, the immunome protein coding genes, the PPIs and the average jackknife gene expression correlation between the immunome protein coding genes.

Network filtering was achieved with the GloSS algorithm [13], which identifies the relevant backbone of a weighted graph while retaining its weight distribution and structure. It uses a global null model to calculate the significance of the links by maintaining the topology of the network while assigning link weights randomly, from the observed weight distribution. The link weights (jackknife correlation coefficients) were multiplied by 100 to allow the p-values to be computed by GloSS. The computed link p-values by GloSS were used to filter the network by removing links in decreasing order of p-value. We monitored the filtering process to make sure that at least a path or connectivity remained between the TCR complex and NF-κB signaling pathways. The steps below represent the filtering procedure:

Step 1: Using GloSS, determine p-value for each edge of the network

Step 2: Select the link with the largest p-value

Step 3: Remove the link from the network

Step 4: Check for presence of connectivity between the NF-κB components and the TCR complex

Step 4.1: If connectivity exists discard the link and go to step 2.

Step 4.2: If connectivity does not exist, return the link to the network and stop.

This procedure was performed for both the NF-κB and the NFAT signaling pathways. Network diameter is the maximum of the shortest paths between the nodes of the network. A connected component is the region of a network in which there is a path connecting all node pairs. We followed changes in the network diameter, the relative size of the largest connected component and the average size of the isolated components [28]. The relative size of the largest component is the number of nodes in the largest component divided by the number of nodes in the whole network. That is, *n*_*rel*_ = *n*/*N*, where, *n*_*rel*_ is the relative size of the largest component, *n* is the number of nodes in the largest component and *N* is the number of nodes in the whole network. These measures were plotted against the fraction of filtered nodes. The ratio,

$$\frac{\mathrm{number}\;\mathrm{of}\;\mathrm{deleted}\;\mathrm{nodes}}{\mathrm{number}\;\mathrm{of}\;\mathrm{nodes}\;\mathrm{in}\;\mathrm{the}\;\mathrm{network}},$$

represents the fraction of the filtered nodes. The igraph package was used to calculate the network scores [50].

### Robustness of the T cell PPI network

Link weight-randomized networks were created by randomizing the weights of a fraction of links, keeping the topology unchanged. The following fractions of links were used to create each of the link weight-randomized networks: 0.1, 0.2, 0.3, 0.4, 0.5, 0.6, 0.7, 0.8 and 0.9. Thirty iterations were performed on each link weight-randomized network. For each iteration, a fraction of links were randomly selected, their weights randomly reassigned, the filtering procedure performed and network topology statistics calculated. Node degree, average path length, betweenness centrality of both the nodes and the links, clustering coefficient of the network, and the intersection between the TPPIN network and the link weight-randomized networks, were calculated. After the iterations for each link weight-randomized network, the average of each of the network topology statistics was retained.

### Gene Ontology term enrichment, over-representation and semantic similarity analysis

The interconnected proteins in the TPPIN were subjected to GO [57] term enrichment analysis. The GO terms for the proteins in the immunome interactome were used as the background. Fisher's exact test of the hypergeometric distribution was calculated and correction for multiple comparisons was performed using the Benjamini-Hochberg procedure [58]. The enrichment analysis was performed with Webgestalt [59]. Semantic similarity between the immunome interactome and the TPPIN was calculated using the clusterSim routine of the GOSemSim library [60] (version 1.18.0) available in R/Bioconductor.

### Analysis of essential genes

We retrieved the human orthologs of the mouse lethality genes from the Mouse Genome Informatics database [29]. A gene was included in the set of lethality genes with the following criteria: phenotype contains the word “lethality”, the type of lethality annotation contains neither “partial” nor “wean”. After removing non-immunome genes and those without the above-mentioned lethality annotations, we calculated the hypergeometric distribution and Fisher’s exact test for significance. Essential genes were retrieved using the biomaRt package in R [61] and visualization of the TPPIN with essential genes was done using Cytoscape 2.8.3.

### Pathway gene mapping

The TPPIN genes were mapped to the KEGG pathways using the KEGG pathway mapper tool [16].

## Competing interests

The authors declare that they have no competing interests.

## Authors’ contributions

GNT contributed towards data acquisition, analysis and interpretation; drafting and writing the manuscript. CO and MV contributed towards conception and design of this work; analysis and interpretation; drafting and writing the manuscript. All authors read and approved the final manuscript.

## Supplementary Material

Additional file 1 **Protein data from the Immunome Knowledge Base and the immune response pathways from KEGG.** This file contains the Entrez-gene identifiers of the genes encoding the immune response proteins from the IKB database and the KEGG immune response pathways listed in Table 1 of the main document. This dataset represents the immunome protein dataset and was used to generate the immunome interactome from PPIs in the iRefIndex database. Click here for file

Additional file 2 **Immunome interactome network figure.** The figure represents the immunome interactome constructed from the immunome protein list of Additional file 1. The figure shows the complex nature of the network and thus cannot be studied by intuition alone. To reduce the complexity of the network the filtering procedure, reported in this study, was performed. Click here for file

Additional file 3 **Immunome interactome table.** This is a table of the PPIs of the immune response proteins of Additional file 1. They were reconstructed from the iRefIndex which is a compendium of PPI data from major PPI databases. Additional filtering was carried out such that only experimentally verified, human, binary PPIs were obtained (see methods). The identifiers are entrez gene identifiers of the genes that code for the immune response genes. Click here for file

Additional file 4 **A summary of the gene expression datasets.** This consists of a summary of all microarray datasets that were used in this study. The datasets were retrieved from NCBI’s GEO and EBI’s ArrayExpress databases. The dataset with asterisk (*) contains 3 experiments conducted on 3 different platforms. The 3 experiments were separated into separate data sets throughout the pre-processing. After pre-processing only samples from the experiment conducted on Affymetrix Human Genome U133A Array were merged with data sets from other experiments. Click here for file

Additional file 5 **Full Gene Ontology analysis results table.** This contains details of the GO term enrichment analysis performed by the Webgestalt web resource. The background of the GO analysis is the immune response proteins. The null hypothesis significance test is the hypergeometric test and the p-values were corrected using the Benjamini–Hochberg procedure. Click here for file
