# Supplementary figures and images for: Identification of core T cell network based on immunome interactome
Source: BMC Syst Biol. 2014 Feb 15;8:17. doi: 10.1186/1752-0509-8-17 (PMC3937033; doi:10.1186/1752-0509-8-17)

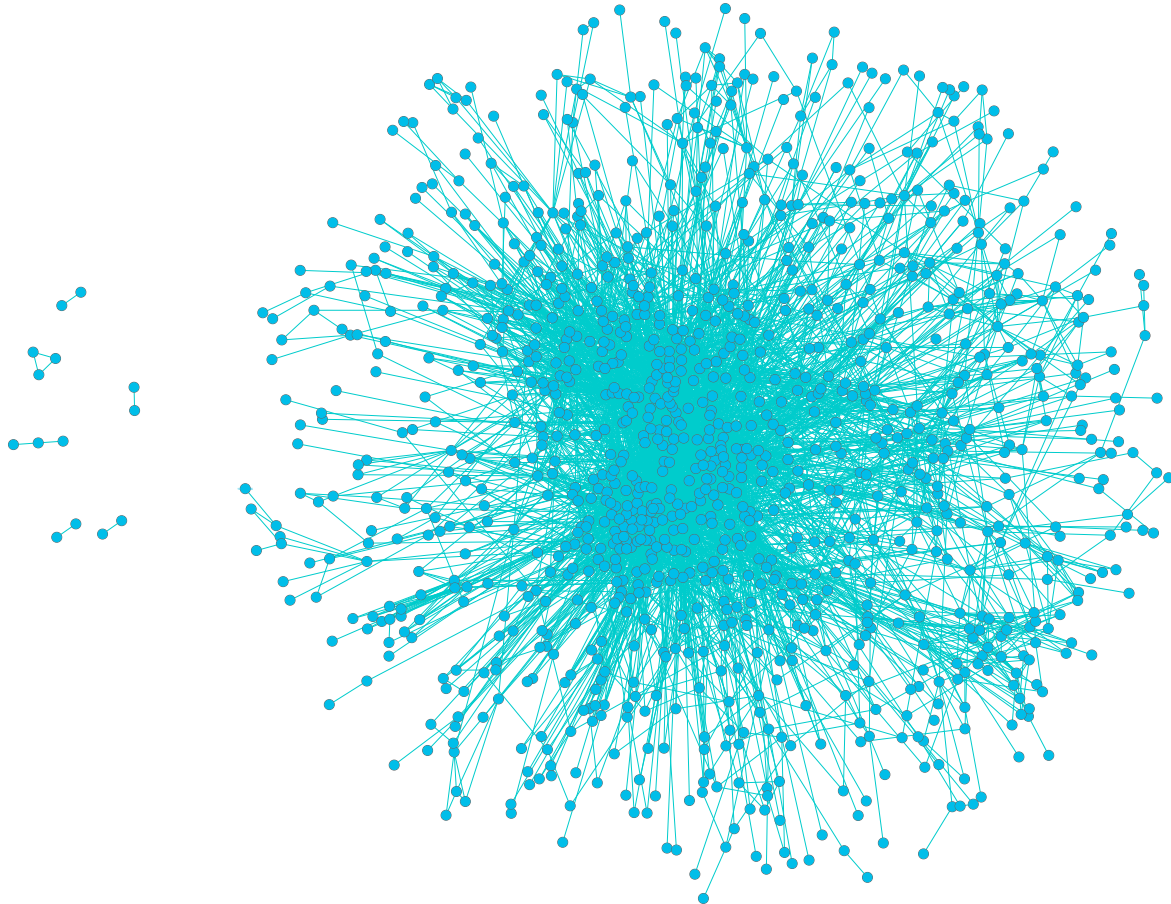

Supplement: Additional file 2 — Immunome interactome network figure. The figure represents the immunome interactome constructed from the immunome protein list of Additional file 1. The figure shows the complex nature of the network and thus cannot be studied by intuition alone. To reduce the complexity of the network the filtering procedure, reported in this study, was performed. [file 1752-0509-8-17-S2.pdf]
